# How effective are common medications: a perspective based on meta-analyses of major drugs

**DOI:** 10.1186/s12916-015-0494-1

**Published:** 2015-10-02

**Authors:** Stefan Leucht, Bartosz Helfer, Gerald Gartlehner, John M. Davis

**Affiliations:** Department of Psychiatry and Psychotherapy, Technische Universität München, Klinikum rechts der Isar, Ismaninger Straße 22, 81675 Munich, Germany; Department for Evidence-based Medicine and Clinical Epidemiology, Danube University Krems, Krems an der Donau, Austria; RTI-International, Research Triangle Park, NC USA; Department of Psychiatry, University of Illinois at Chicago, Chicago, IL USA

**Keywords:** Absolute risk or response difference, Common medications, Drug classes, Drug efficacy, Mean difference, Medication efficacy, Meta-analysis, Percentage response ratio, Pharmacological interventions, Standardized mean difference, Schizophrenia, Depression

## Abstract

The vastness of clinical data and the progressing specialization of medical knowledge may lead to misinterpretation of medication efficacy. To show a realistic perspective on drug efficacy we present meta-analyses on some of the most commonly used pharmacological interventions. For each pharmacological intervention we present statistical indexes (absolute risk or response difference, percentage response ratio, mean difference, standardized mean difference) that are often used to represent efficacy. We found that some of the medications have relatively low effect sizes with only 11 out of 17 of them showing a minimal clinically important difference. Efficacy was often established based on surrogate outcomes and not the more relevant patient-oriented outcomes. As the interpretation of the efficacy of medication is complex, more training for physicians might be needed to get a more realistic view of drug efficacy. That could help prevent harmful overtreatment and reinforce an evidence-based, but personalized medicine.

**Electronic supplementary material:**

The online version of this article (doi:10.1186/s12916-015-0494-1) contains supplementary material, which is available to authorized users.

## Background

Medicine is becoming so highly specialized and the clinical literature is growing so fast, that few doctors let alone the lay public have a working knowledge of the detailed evidence on drugs outside their specialty [1]. This is despite the fact that clinicians must often evaluate comparative risks and benefits of treatments for patients with multiple maladies. Studies show that decision making can be distorted by various cognitive biases such as a physician’s tendency to remember dramatically successful cases and forget ones that failed or to misinterpret the statistical indices used in clinical trials and meta-analyses [2]. This may lead the physician to overestimate the efficacy of treatments, which in turn may be one of the causes of harmful overtreatment [3].

### Common pharmacological treatments

We would like to present a realistic perspective on the general efficacy of common pharmacological treatments. Following the general methods of a previous overview of reviews [4], we identified systematic reviews of randomized controlled trials with meta-analysis comparing drugs used in specific therapy types with placebo. We included 20 most common therapy types as measured by the number of on-therapy patients in the US, according to the IMS Institute for Healthcare Informatics [5]. For each therapy type listed there we identified primary pharmacological treatments and their primary indications (as suggested by the IMS review and verified by national and international treatment guidelines). Then using PubMed we searched (last search: 5 August 2014, see Additional file 1) for the broadest and most recent meta-analysis on that treatment. If possible, we included meta-analyses on monotherapy rather than combination therapy, on all patients rather than a sub-group of patients (for example, we preferred reviews on all age groups, over ones restricted to adults or children) and on broad drug classes rather than narrow ones or single drugs (for example, we preferred a meta-analysis on all antihypertensive drugs, over ones on ACE inhibitors or enalapril). If a meta-analysis on the whole therapy type (for example, any narcotic) was not available, we included a frequently used example (for example, oxycodone + paracetamol, which is the most frequently used painkiller according to the IMS report for which we found a meta-analysis fulfilling our inclusion criteria). For a more detailed description of our methods, please refer to the protocol (see Additional file 2).

### Measures of medication efficacy

**Fig. 1:**
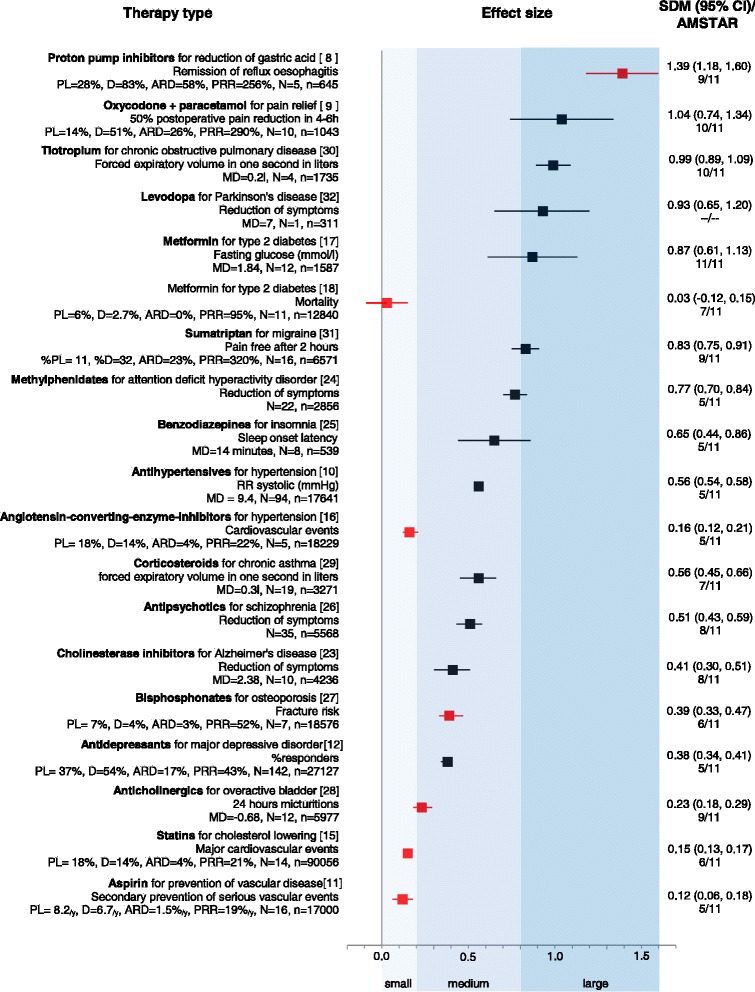
Summary of effect sizes for common pharmacological treatments. The figure presents primary pharmacological intervention for a given therapy type, the primary outcome, descriptive statistics and efficacy measures. Effect sizes are expressed as standardized mean difference with corresponding confidence intervals on the right side and the AMSTAR score below. The graph in the middle shows a ranking of effect sizes according to Cohen: small effect size is no bigger than 0.2; medium effect size is around 0.5; and large effect sizes are bigger than 0.8. Marked with red color are outcomes that can be objectively measured and are patient-oriented [8–12, 15–18, 23–32]. The following drugs listed by the IMS Institute report were not included in the figure: thyroid preparations (no meta-analysis was found); anti-epileptics (no meta-analysis on monotherapy was found because current antiepileptic trials are add-on); hormonal contraceptives for birth control (no “disease” as an indication); and alpha-adrenergic antagonists for benign prostate hyperplasia (no SMD was provided or calculable). All values are statistically significant (except mortality for metformin). All additional confidence intervals can be obtained from the authors upon request. AMSTAR, a measurement scale for the assessment of the methodological quality of systematic reviews; ARD, absolute risk or response difference; CI, confidence interval; D, percentage of patients with the outcome in the drug group; MD, mean difference in original units; n, number of participants; N, number of trials; PL, percentage of patients with the outcome in the placebo group; PRR, percentage response ratio; SMD, standardized mean difference

Figure 1 lists examples of medications used primarily in the 20 most common therapy types together with a number of statistical indices. Here we explain how these measures are calculated and give some examples:

1. Absolute risk or response difference (ARD) is the risk or percentage of responders in group B subtracted from the risk or percentage of responders in group A. For example, mortality was 2 % for drug treatment and 4 % for placebo, which gives an ARD = |-2 %|. For responder rates, if 45 % of patients responded in the drug group and 30 % in the placebo group, the ARD is 15 %.
2. Percentage response ratio (PRR) is the percentage of responders in group A divided by the percentage responders in group B. For example, if 45 % of participants responded to drug treatment in group A and 30 % to placebo in group B, the PRR is 50 %, because 0.45/0.3 = 1.5. This means that there were 50 % more responders in group A compared to group B.
3. Mean difference (MD) is the mean from group B subtracted from the mean in group A. For example, if the mean total sleep time at the end of treatment in the drug group was 5 hours and 10 minutes and in the placebo group 4 hours and 55 minutes, the MD is 15 minutes.
4. Standardized mean difference (SMD) is the mean from group B subtracted from the mean in group A and divided by the pooled standard deviation (SD). For example, if the average weight of participants at the end of treatment was 79 kg in the drug group and 83 kg in the placebo group and the pooled SD was 8 kg, the SMD is 0.5.

Effect sizes at Fig. 1 are expressed graphically as SMDs and are ranked as “small” (0.2), “medium” (around 0.5) or “large” (above 0.8) [6]. We also present the percentage of responders in the drug and placebo group and, if appropriate, the number of trials (N) and patients (n) for each meta-analysis, as well as the AMSTAR score, which is a measure of methodological quality of systematic reviews [7].

### The efficacy of common medications

Differences larger than one standard deviation (that is, SMD >1) between the drug and placebo groups are uncommon, examples being proton pump inhibitors for reflux esophagitis [8] or oxycodone plus paracetamol for postoperative pain [9]. For many other medications the effect sizes were much smaller. For example, antihypertensive drugs reduced systolic and diastolic blood pressure by only 10 mmHg and 5 mmHg, respectively [10], the ARD between aspirin and placebo for primary prevention of cardiovascular events was only 0.07 % per year [11], and the ARD for antidepressants and placebo for major depressive disorder was 17 % [12].

For an outcome affecting quality of life, ½ of a standard deviation is considered to be a minimal clinically important difference [13]. Out of 17 common pharmacological treatments examined, only 11 met this threshold. In four of them efficacy was represented by surrogate outcomes, such as diastolic blood pressure or fasting plasma glucose, and not patient-oriented outcomes, such as pain, mortality or adverse events. Therefore, patients might not have experienced substantial benefits related to their well-being and quality of life after therapy with some of these drugs. Moreover many of the included meta-analyses had a low methodological quality as represented by median AMSTAR score of 7/11 (interquartile range 5 to 9).

### Surrogate outcomes versus patient-oriented outcomes

Figure 1 also illustrates that surrogate outcomes often show dramatic effects, while the effects on patient-oriented outcomes are much smaller. For example, statins reduce cholesterol by 30 % on average [14]. However, high cholesterol alone does not directly produce pain or disability. For long-term consequences, such as cardiovascular events and mortality, the effects are smaller (ARD between statins and placebo of 4 % for cardiovascular events and 1.2 % for mortality within 5 years [15]). In hypertension, medium effect sizes for reductions of hypertension [10] lead to comparatively small reductions of cardiovascular events [16], and metformin strongly reduces glucose [17], but there is no evidence of a reduction in mortality [18]. Among the seven outcomes that can be both objectively measured and are patient-oriented (marked in red color in Fig. 1) only one shows a big effect size (remission of reflux esophagitis by proton pump inhibitors [8]).

### Statistical indices can be misleading

In general, relative risk reductions suggest larger differences than ARDs. For example, statins reduced the number of patients with major cardiovascular events from 18 % to 14 % [15]. The relative risk reduction of 21 % (100 % - (14 %/18 %) = 21 %) is more impressive than the ARD of 4 % (14 % - 18 % = |-4 %|). Findings consistently show that a mere reporting of a relative risk reduction can be misleading, because many clinicians will interpret it as an absolute difference [19].

### Limitations

There are many limitations in an overview of meta-analyses [4]. For example, the meta-analyses differed in methods and publication year. We preferred reviews of drug classes which may obscure superiorities of single drugs. Many outcomes may accumulate over time if the studies had longer durations. For example, the evidence on mortality reduction by statins is based on 5-year studies, but the effect could get larger if patients took them for 20 years. Or a patient with depression may have ten episodes in his life which could be reduced by medication to five [20]. Finally, whether the increment of improvement by a drug is important depends on many factors, such as the seriousness of the disease, side-effects, cost and, most importantly, the short- and long-term outcome in question. For mortality, the “baseline risk” (that is, mortality in the no-treatment group) is often low, leading to a relatively low maximally possible absolute risk reduction. For example, within 5 years without treatment only 9.7/100 participants with hypercholesterolemia died [15], limiting the maximally possible absolute mortality reduction to 9.7 %. Nevertheless, since mortality is such an important outcome, even a small reduction can be clinically meaningful. In other words, a large effect size for a transitory rash is less important than a small reduction of death. For all these reasons, this article is only a perspective and not a full review of the evidence for every possible aspect.

## Conclusions

We feel that we need to be more realistic about drug efficacy. Doctors may believe that all patients respond to drugs and none to placebo, but neither statement is true because there is no ideal drug and many disorders remit spontaneously due to their natural course. Our preference for black or white over shades of grey is convenient but it can offer only a “false clarity” [21]. The psychologist Daniel Kahneman received the Nobel Prize in economics for research on cognitive bias and decision making, seen in the context of an initial perception of an idea, which takes place in less than a second versus the more logical thinking through of ideas, which often takes hours or days [22]. The initial rapid intuitions can be biased by many factors such as recency, frequency and vividness of prior personal experiences, but does not take into account statistics very well. Pharmaceutical company advertising takes full advantage of this. We feel these quantitative benchmarks will help clinicians learn how to interpret the latest drug findings and reflect on their limitations. We do not strive to therapeutic nihilism, but rather believe that drug data is complex and requires thoughtful consideration regarding which medications and therapies are best suited for certain situations and patients.

## Additional files

Additional file 1:
**Details and results of the search.** (PDF 257 kb) Additional file 2:
**Study protocol.** (PDF 161 kb)

## Abbreviations

AMSTAR: A measurement scale for the assessment of the methodological quality of systematic reviews
ARD: Absolute response or risk difference
CI: Confidence interval
D: Percentage of patients with the outcome in the drug group
MD: Mean difference in original units
n: Number of participants
N: Number of trials
PL: Percentage of patients with the outcome in the placebo group
PRR: Percentage response ratio
SMD: Standardized mean difference
