# Supplementary material for: How effective are common medications: a perspective based on meta-analyses of major drugs
Source: BMC Med. 2015 Oct 2;13:253. doi: 10.1186/s12916-015-0494-1 (PMC4592565; doi:10.1186/s12916-015-0494-1)
Supplement: Additional file 1: — Details and results of the search. (PDF 257 kb) [file 12916_2015_494_MOESM1_ESM.pdf]

## Additional file 1

## Details and results of the search

PubMed search syntax, PRISMA diagrams for all therapy types and PICO questions for the included meta-analyses. The update search was conducted in August 2014.

**[1] Hypertension (antihypertensive drugs)**
(„Hypertension"[Mesh]) AND "Meta-Analysis"[Publication Type]

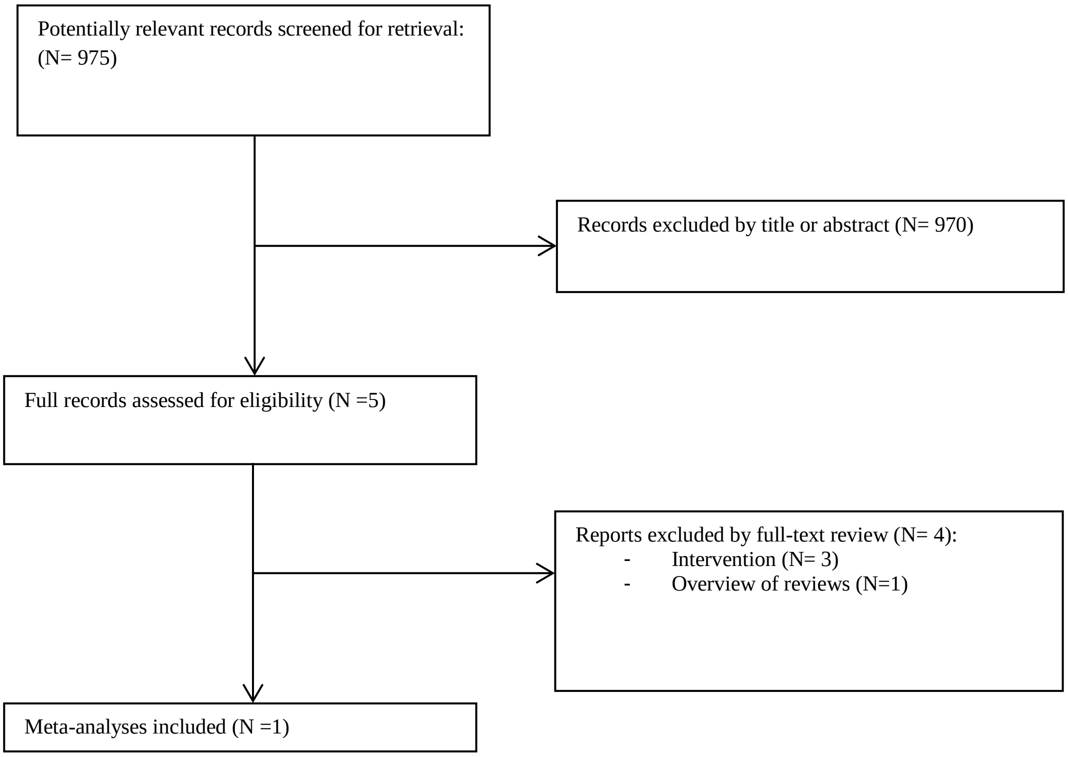


Included study 1:
Law M. Morris JK. Jordan R. Wald N. Headaches and the treatment of blood pressure: results from a meta-analysis of 94 randomized placebo-controlled trials with 24.000 participants. Circulation 2005;112:2301-6.

PICO questions:
People: adults
Intervention: 4 different classes of blood pressure–lowering drugs (thiazides, beta-blockers, ACE inhibitors, and angiotensin II receptor antagonists) in fixed doses
Comparison: placebo
Outcome: reductions in systolic and diastolic blood pressures, reduction in headaches

Included study 2 (selected post-hoc from the reports excluded by full-text review):
Turnbull, F. Blood Pressure Lowering Treatment Trialists, Collaboration. Effects of different blood-pressure-lowering regimens on major cardiovascular events: results of prospectively-designed overviews of randomized trials. Lancet 2003;362:1527-35.

PICO questions:
People: adults
Intervention: treatment strategies based on different drug classes (angiotensin-converting-enzyme inhibitors, calcium antagonists, angiotensin-receptor blockers, diuretics, beta blockers)
Comparison: placebo
Outcome: risks of total major cardiovascular events

**[2] Cholesterol (statins)**

("lipid regulating agents"[MeSH Terms]) AND " Meta-Analysis "[Publication Type]

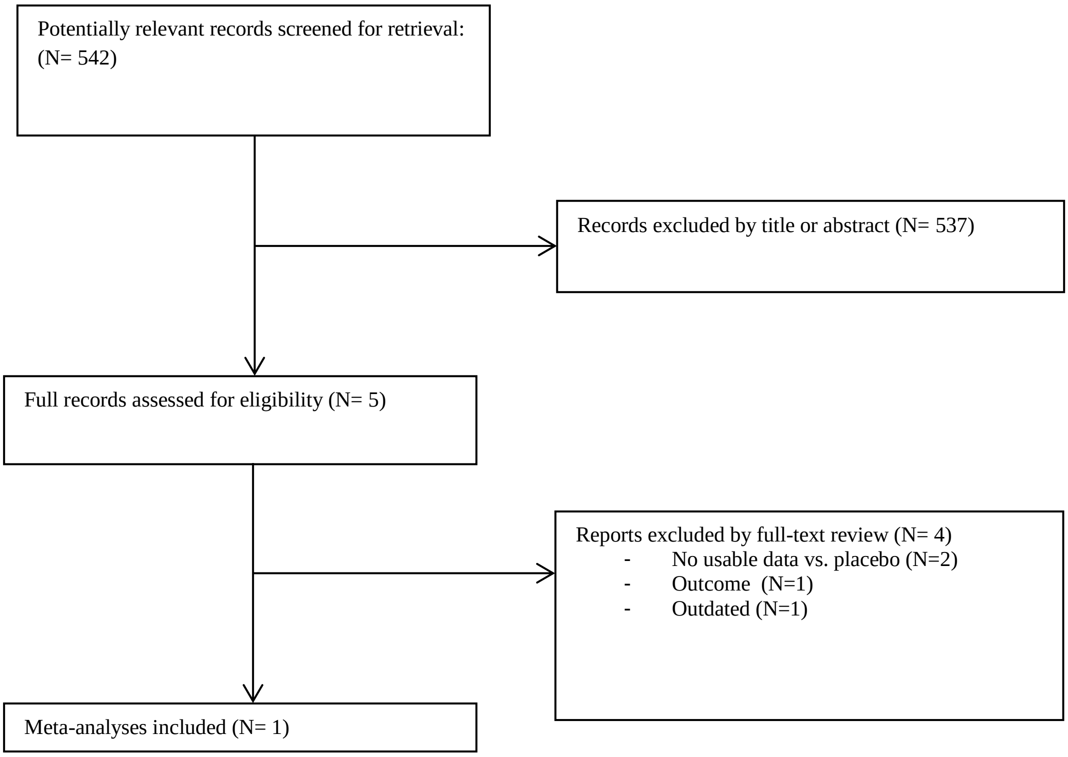


Included study:
Baigent C. Keech A. Kearney PM. et al. Efficacy and safety of cholesterol-lowering treatment: prospective meta-analysis of data from 90.056 participants in 14 randomised trials of statins. Lancet 2005;366:1267-78.

PICO questions:

| | |
|---|---|
| People: | all |
| Intervention: | statin therapy |
| Comparison: | placebo, no treatment, usual care |
| Outcome: | absolute LDL cholesterol difference, all-cause mortality, coronary heart disease mortality, and non- coronary heart disease mortality, major cardiovascular events |

**[3] Antidepressants (major depressive disorder)**
(("depressive disorder"[MeSH Terms]) OR "antidepressive agents"[MeSH Terms]) AND "meta analysis"[Publication Type]

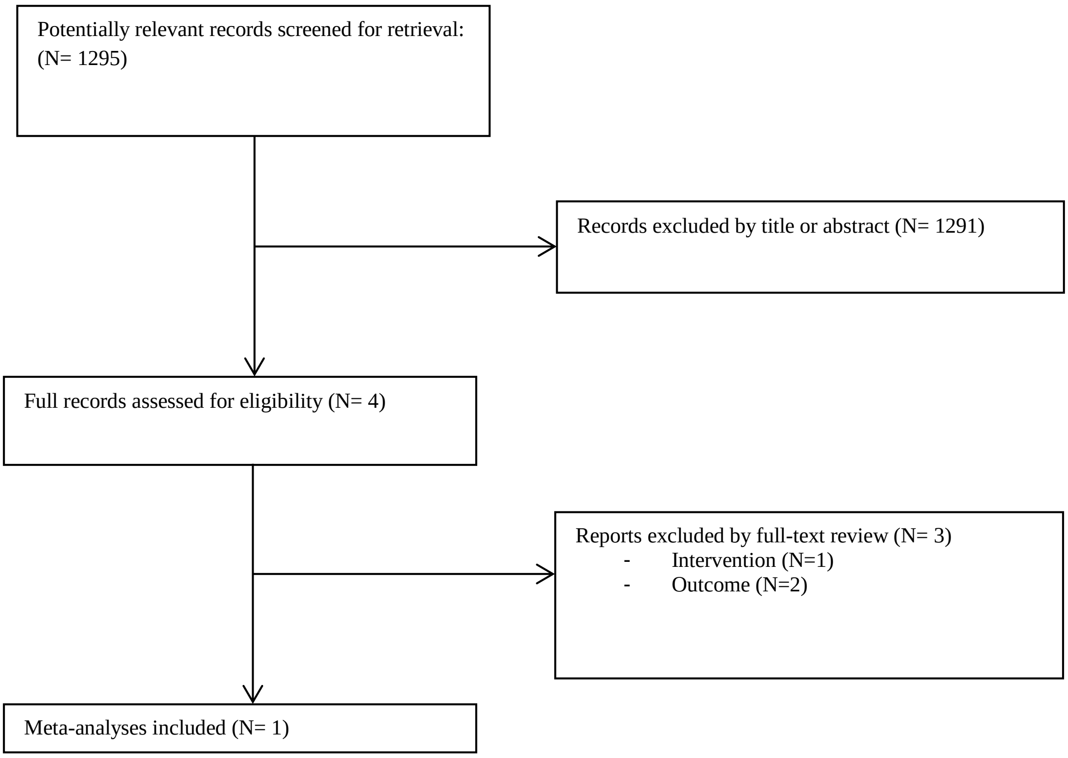


<u>Included study:</u>
Undurraga J. Baldessarini RJ. Randomized. placebo-controlled trials of antidepressants for acute major depression: thirty-year meta-analytic review. Neuropsychopharmacology. 2012 Mar;37(4):851-64. doi: 10.1038/npp.2011.306. Epub 2011 Dec 14

<u>PICO questions:</u>
People: adults in an acute major depressive episode
Intervention: antidepressants
Comparison: placebo
Outcome: reduction in initial depression rating-scale scores

**[4] Anti-Ulcerants (proton pump inhibitors)**

("proton pump inhibitors"[MeSH Terms]) AND "meta analysis"[Publication Type]

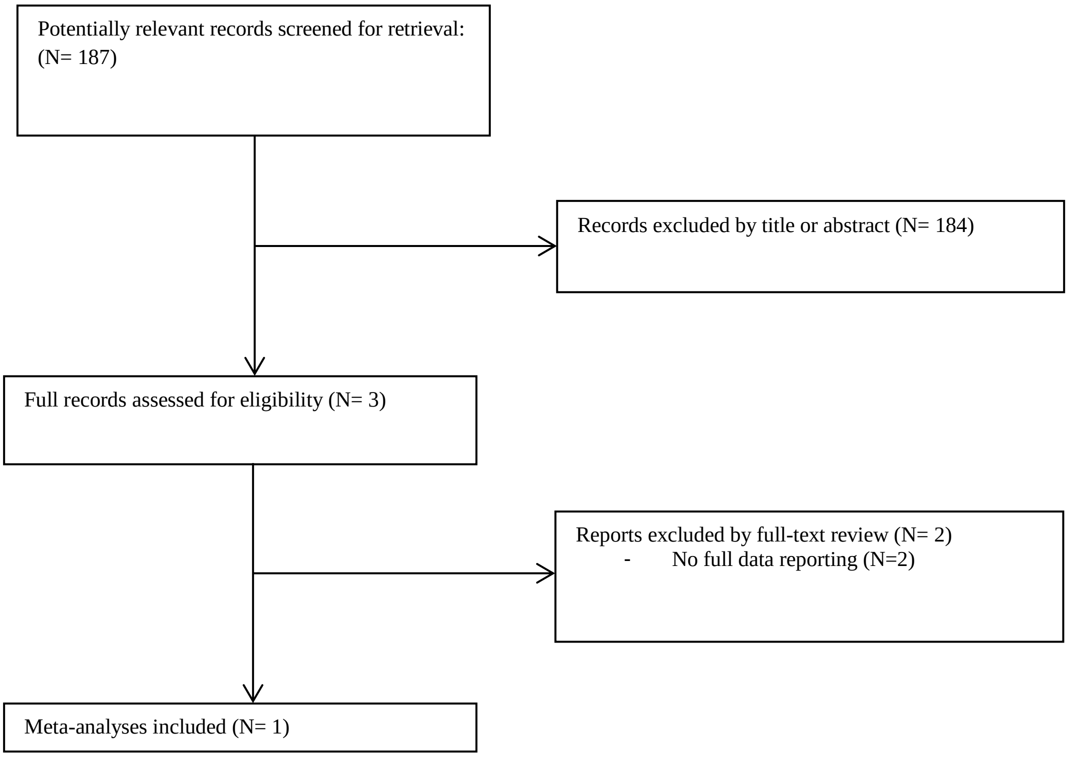


<u>Included study:</u>
Moayyedi P. Santana J. Khan M. Preston C. Donnellan C. Medical treatments in the short term management of reflux oesophagitis. Cochrane Database Syst Rev 2007:CD003244

<u>PICO questions:</u>

| | |
|---|---|
| People: | adults who had reflux oesophagitis diagnosed at endoscopy |
| Intervention: | proton pump inhibitors, H2 receptor antagonists, prokinetic therapy, sucralfate |
| Comparison: | against each other and placebo |
| Outcome: | proportion of patients who had reflux oesophagitis symptoms or oesophagitis persisting |

**[5] Narcotics (analgesics for postoperative pain)**
("narcotics"[MeSH Terms]) AND "meta analysis"[Publication Type]

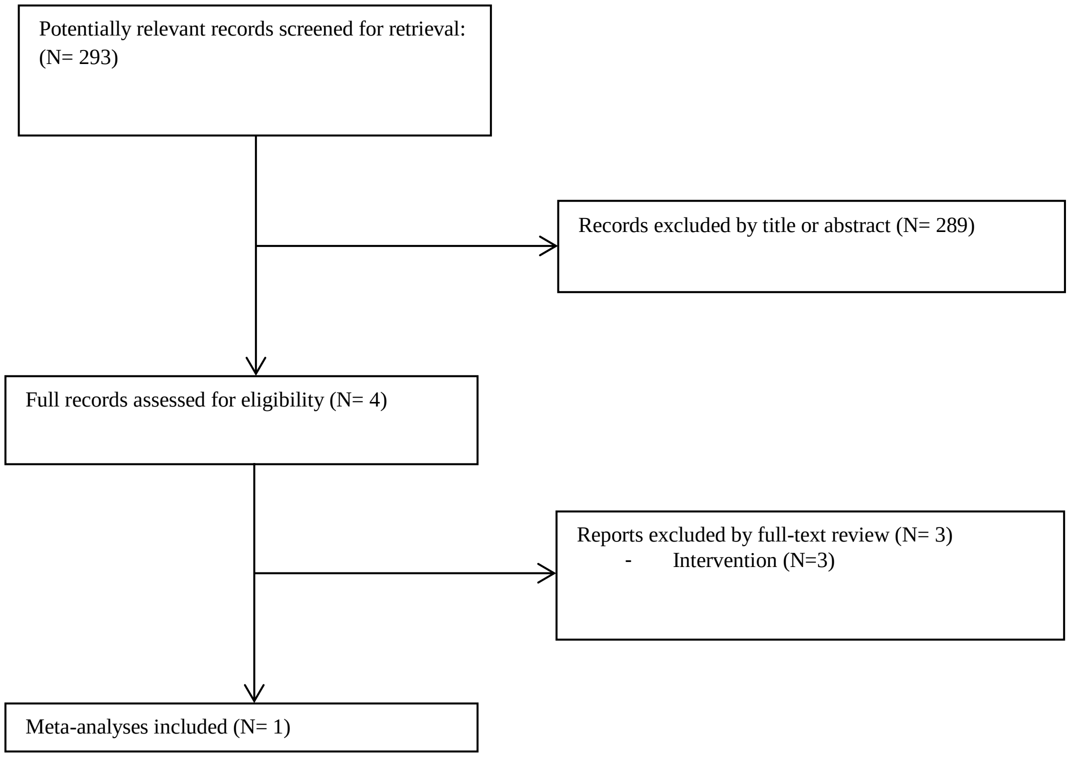


Included study:
Moore RA. Derry S. McQuay HJ. Wiffen PJ. Single dose oral analgesics for acute postoperative pain in adults. Cochrane Database Syst Rev 2011:CD008659.

PICO questions:

| | |
|---|---|
| People: | adults with acute postoperative pain |
| Intervention: | single dose oral analgesics |
| Comparison: | placebo |
| Outcome: | at least 50% maximum pain relief over four to six hours |

**[6] Antidiabetes (metformin, GLP-1 analogues, DPP4-Inhibitors, SGLT2 inhibitors)**
("hypoglycemic agents"[MeSH Terms]) AND "meta analysis"[Publication Type]

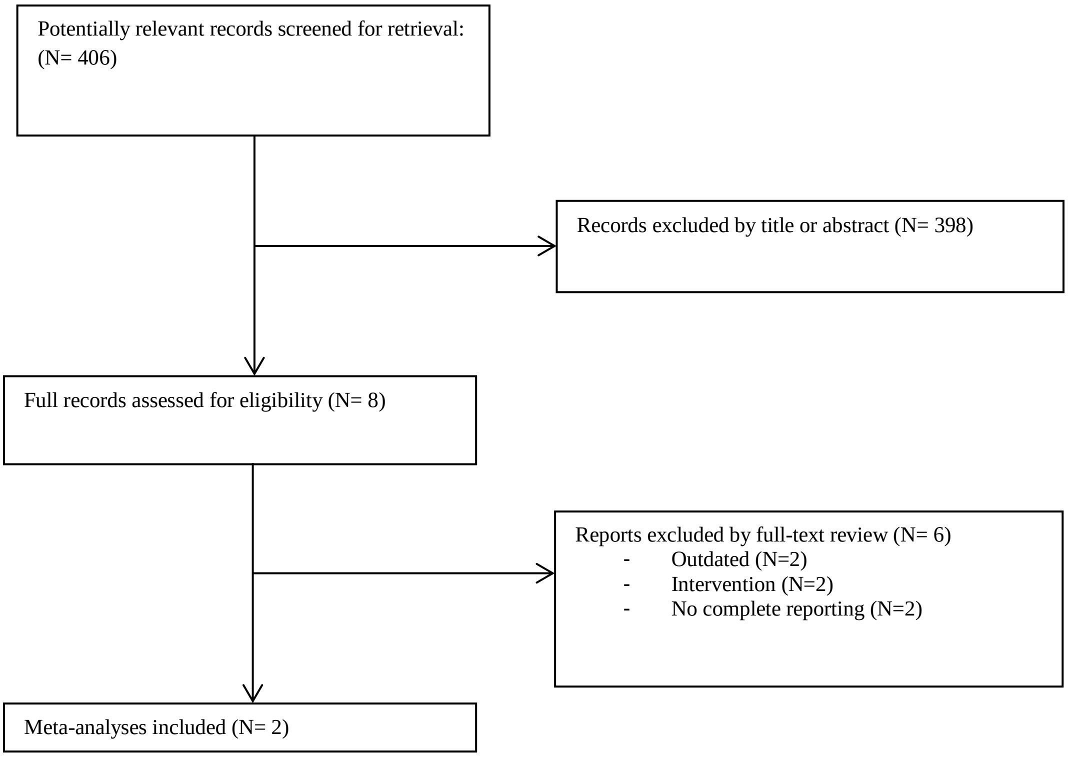


Included study 1:
Boussageon R. Supper I. Bejan-Angoulvant T. et al. Reappraisal of metformin efficacy in the treatment of type 2 diabetes: a meta-analysis of randomised controlled trials. PLoS medicine 2012;9:e1001204.

PICO questions:

| | |
|---|---|
| People: | people with type 2 diabetes |
| Intervention: | metformin |
| Comparison: | no treatment, placebo, diet |
| Outcome: | all-cause mortality and cardiovascular death |

Included study 2:
Saenz A. Fernandez-Esteban I. Mataix A. Ausejo M. Roque M. Moher D. Metformin monotherapy for type 2 diabetes mellitus. Cochrane Database Syst Rev 2005:CD002966

PICO questions:

| | |
|---|---|
| People: | people with type 2 diabetes |
| Intervention: | metformin |
| Comparison: | placebo, diet, or another pharmaceutical intervention |
| Outcome: | mortality, morbidity, quality of life, glycaemic control, body weight, lipid levels, blood pressure, insulinaemia, and albuminuria |

**[7] Thyroid (thyroid preparations for hypothyroidism)**

("hypothyroidism"[MeSH Terms]) AND "meta analysis"[Publication Type]

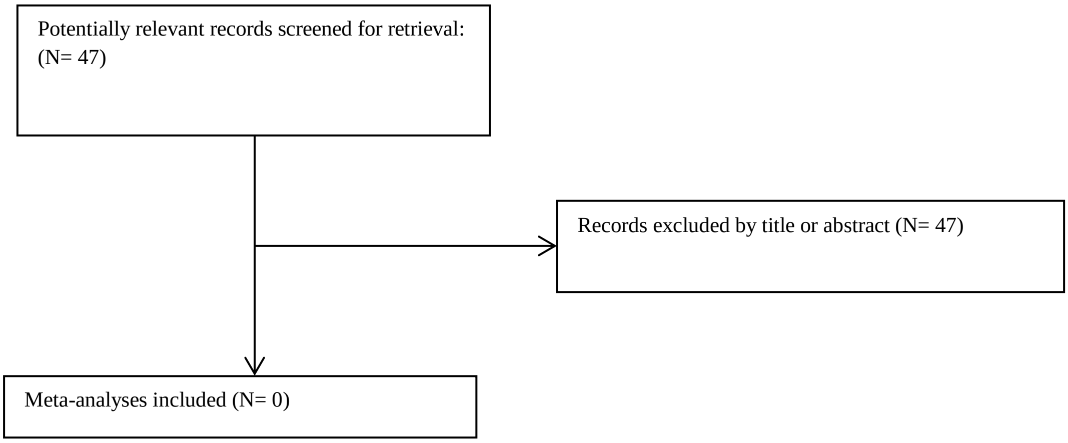


**[8] Anti-Epileptics (epileptic seizures)**
(("epilepsy"[MeSH Terms]) AND "anticonvulsants"[MeSH Terms]) AND "meta analysis"[Publication Type]

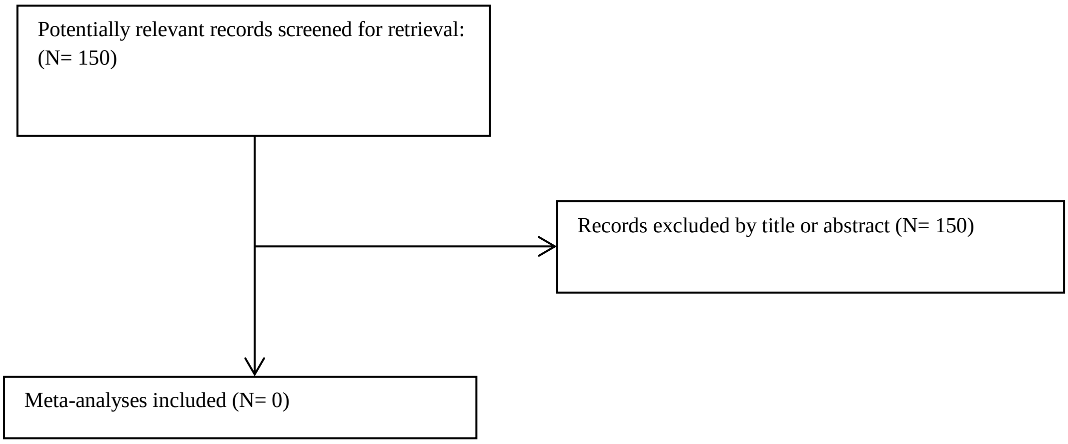


**[9] Contraceptives (hormonal contraceptives for birth control)**
Excluded: no “disease” as an indication

**[10] Respiratory (asthma, COPD)**

(("asthma"[MeSH Terms]) OR "pulmonary disease, chronic obstructive"[MeSH Terms]) AND "meta analysis"[Publication Type]

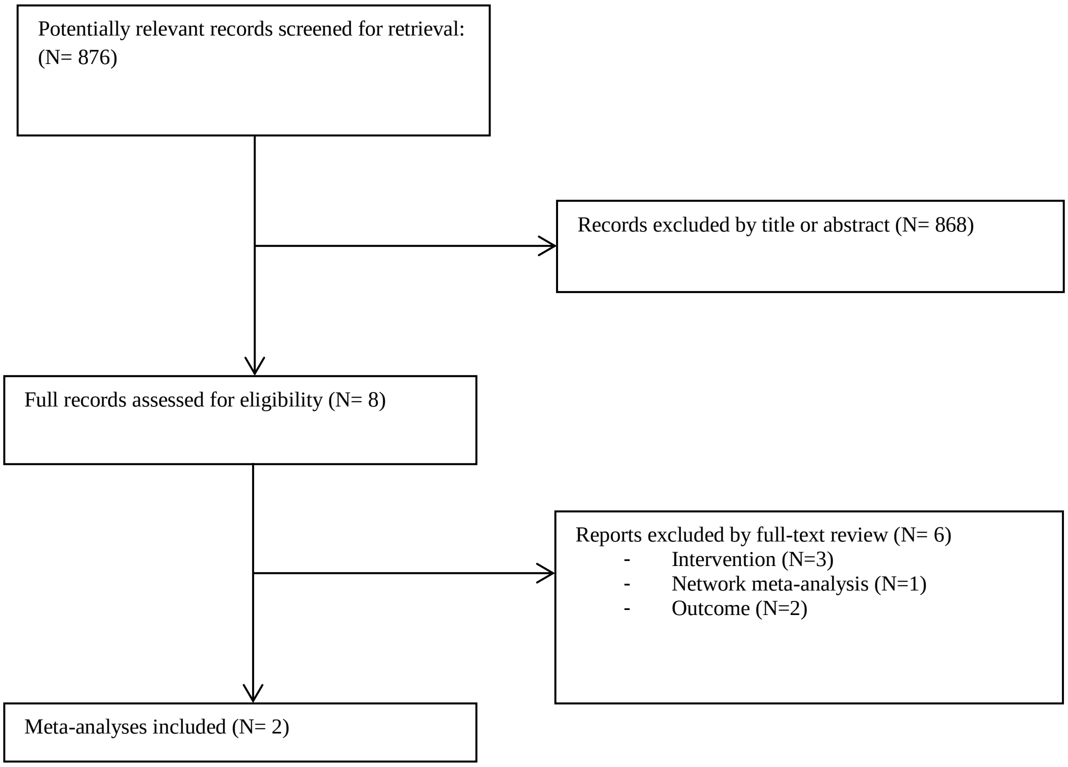


Included study 1:
Barr RG. Bourbeau J. Camargo CA. Ram FS. Tiotropium for stable chronic obstructive pulmonary disease: A meta-analysis. Thorax 2006;61:854-62.

PICO questions:
People: people with stable chronic obstructive pulmonary disease
Intervention: tiotropium,
Comparison: placebo, ipratropium bromide, or long acting Beta2 agonists
Outcome: clinical events, symptom scales, pulmonary function, and adverse events

Included study 2:
Sin DD. Man J. Sharpe H. Gan WQ. Man SF. Pharmacological management to reduce exacerbations in adults with asthma: a systematic review and meta-analysis. JAMA 2004;292:367-76

PICO questions:
People: adults with chronic asthma
Intervention: inhaled corticosteroids, long-acting beta2 agonists, leukotriene pathway modifiers/ receptor antagonists, and anti-IgE therapies
Comparison: placebo, each other
Outcome: exacerbations and/or forced expiratory volume in 1 second

**[11] Antiplatelets/anticoagulants (acetylsalicylic acid)**
((antiplatelet agents[MeSH Terms]) OR anticoagulant agents[MeSH Terms]) AND "meta analysis"[Publication Type]

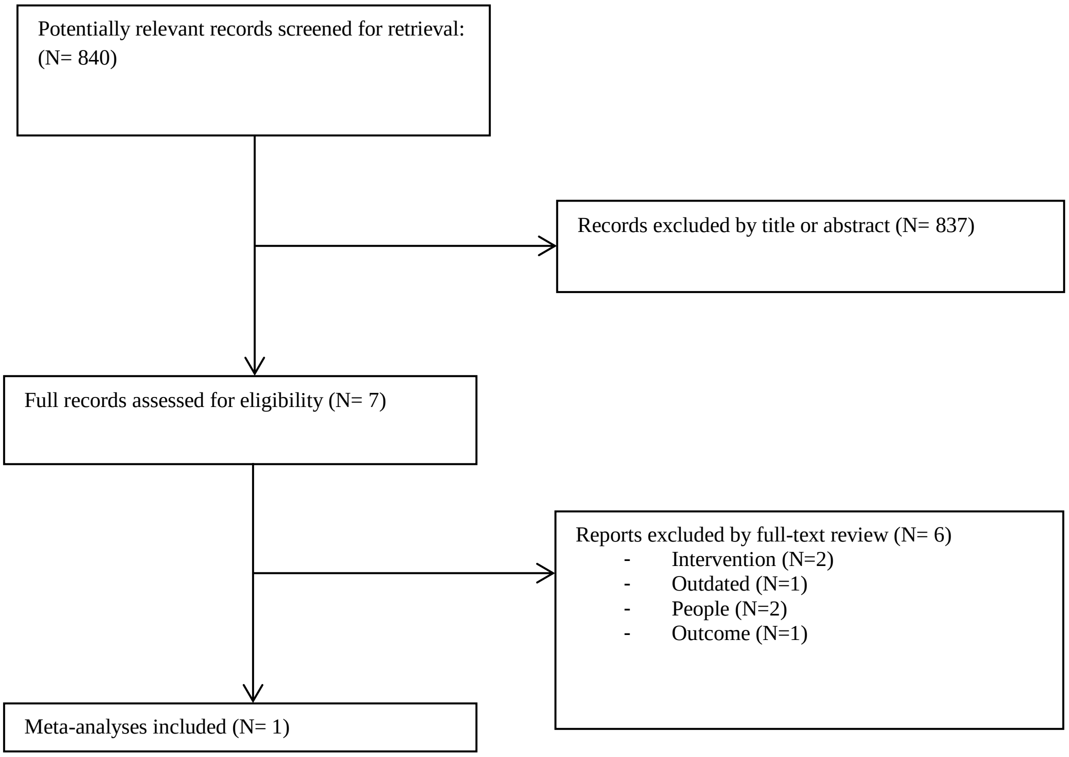


<u>Included study:</u>
Baigent C. Blackwell L. Collins R. Emberson J. Godwin J. Peto R et al. Aspirin in the primary and secondary prevention of vascular disease: collaborative meta-analysis of individual participant data from randomised trials. Lancet 2009;373:1849-60.

<u>PICO questions:</u>
People: non-diabetic participants without occlusive disease at entry
Intervention: long-term aspirin
Comparison: no aspirin (with no other antiplatelet drug in either group), including placebo
Outcome: serious vascular events (myocardial infarction, stroke, or vascular death) and major bleeds

**[12] ADHD (Amphetamines, Methylphenidates)**
("attention deficit disorder with hyperactivity"[MeSH Terms]) AND "meta analysis"[Publication Type]

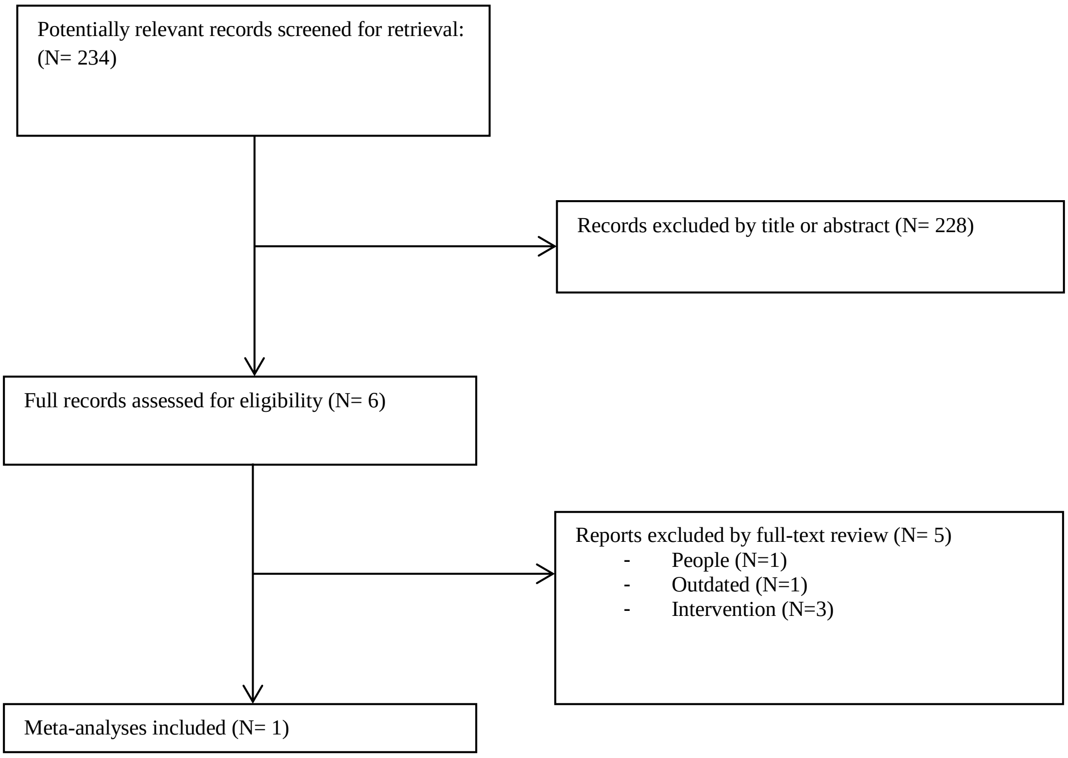


<u>Included study:</u>
Faraone SV1. Buitelaar J. Comparing the efficacy of stimulants for ADHD in children and adolescents using meta-analysis. Eur Child Adolesc Psychiatry. 2010 Apr;19(4):353-64. doi: 10.1007/s00787-009-0054-3. Epub 2009 Sep 10

<u>PICO questions:</u>
People: children and adolescents with ADHD
Intervention: amphetamine or methylphenidate
Comparison: placebo
Outcome: reduction in ADHD symptoms

**[13] Insomnia (Benzodiazepines)**
(insomnia[MeSH Terms]) AND "meta analysis"[Publication Type]

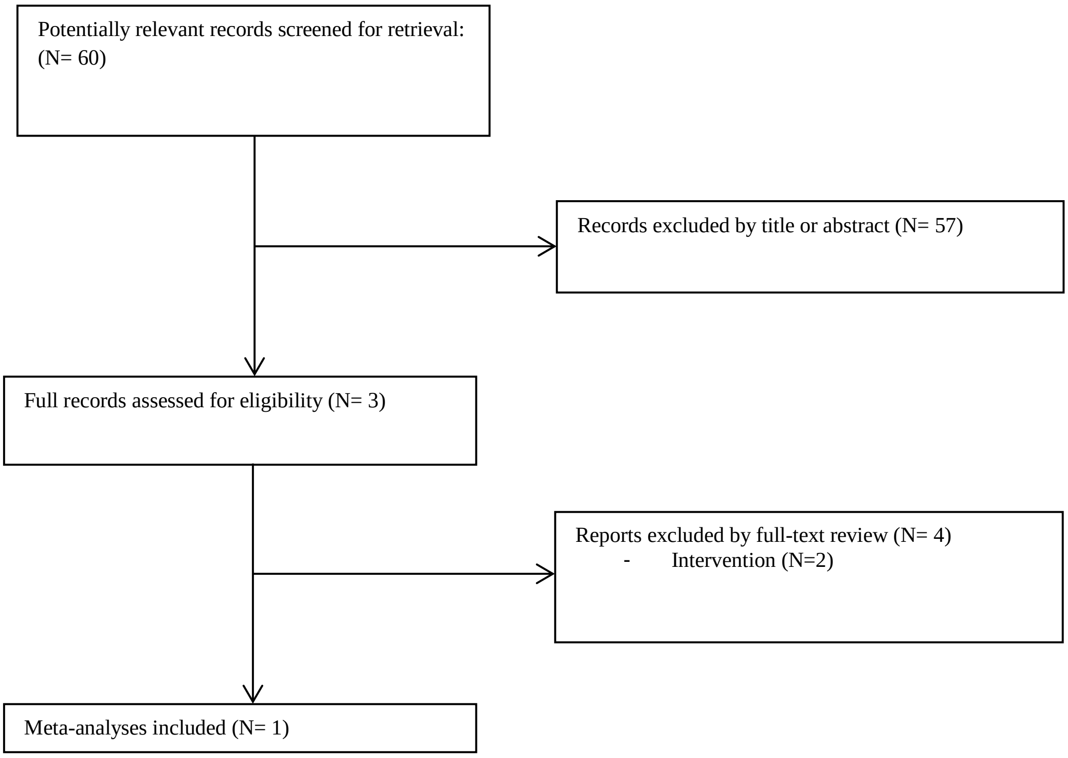


<u>Included study:</u>
Holbrook AM. Crowther R. Lotter A. Cheng C. King D. Meta-analysis of benzodiazepine use in the treatment of insomnia. Canadian Medical Association Journal. 2000;162(2):225-233

<u>PICO questions:</u>
People: adults with insomnia
Intervention: benzodiazepines
Comparison: placebo or another active agent
Outcome: sleep latency, total sleep time, adverse effects

**[14] Benign Prostate Hyperplasia (a-adrenergic blockers, 5-alpha-reductase inhibitors)**
(("adrenergic alpha antagonists"[MeSH Terms]) OR "5 alpha reductase inhibitors"[MeSH Terms]) AND "meta analysis"[Publication Type]

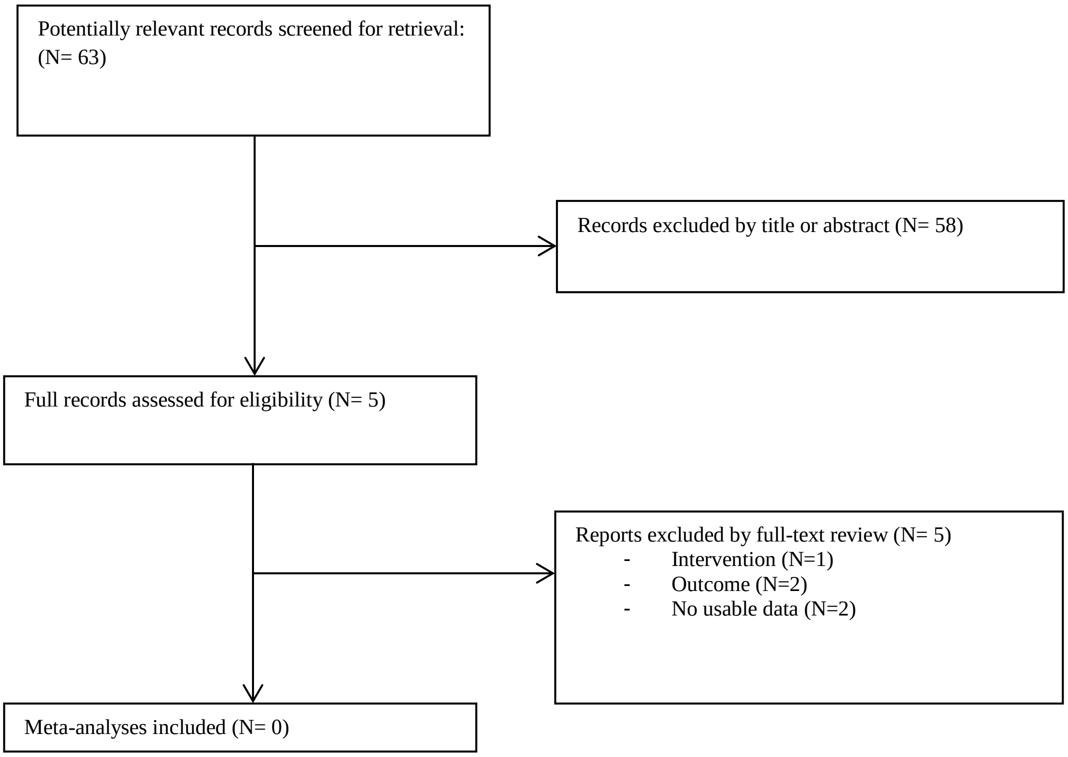

**[15] Antipsychotics (schizophrenia)**
(("antipsychotic agents"[MeSH Terms]) AND "schizophrenia"[MeSH Terms]) AND "meta analysis"[Publication Type]

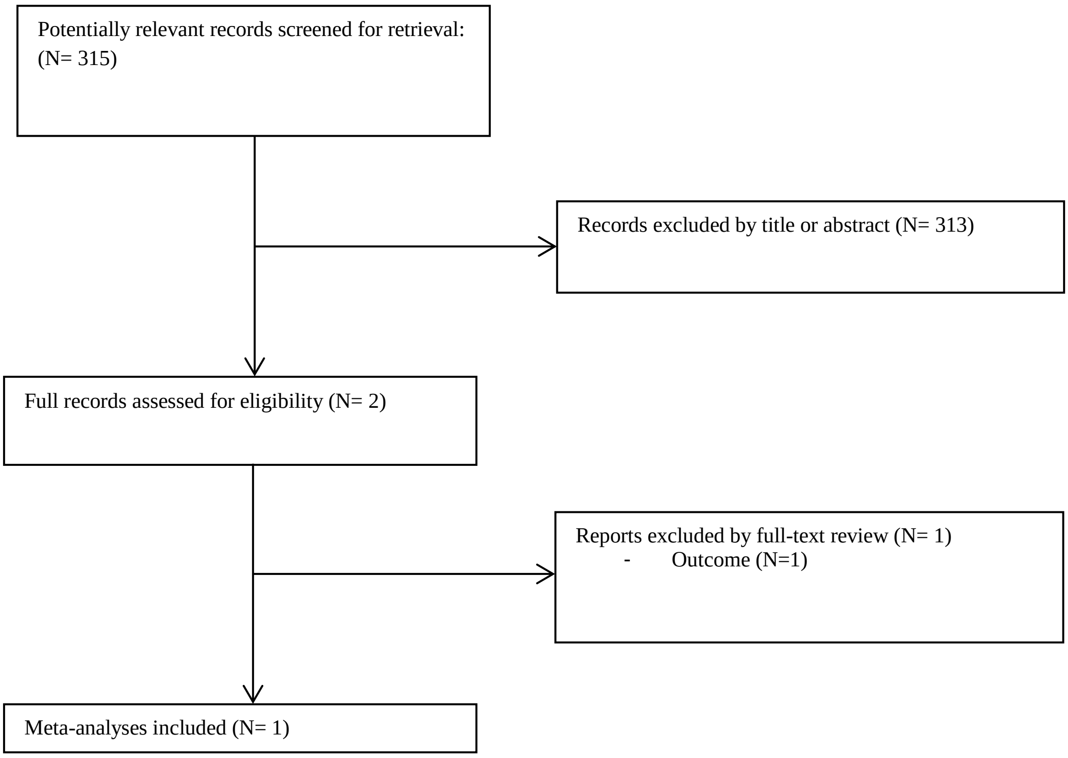


<u>Included study:</u>
Leucht S. Arbter D. Engel RR. Kissling W. Davis JM. How effective are second generation antipsychotic drugs? A meta-analysis of placebo-controlled trials.Molecular Psychiatry. 2009;14 (4):429-447.

<u>PICO questions:</u>

| | |
|---|---|
| People: | schizophrenic patients |
| Intervention: | second-generation antipsychotic drugs |
| Comparison: | placebo |
| Outcome: | reduction of schizophrenic symptoms |

**[16] Osteoporosis (bisphosphonates)**
((diphosphonates[MeSH Terms]) AND osteoporosis[MeSH Terms]) AND "meta analysis" [Publication Type]

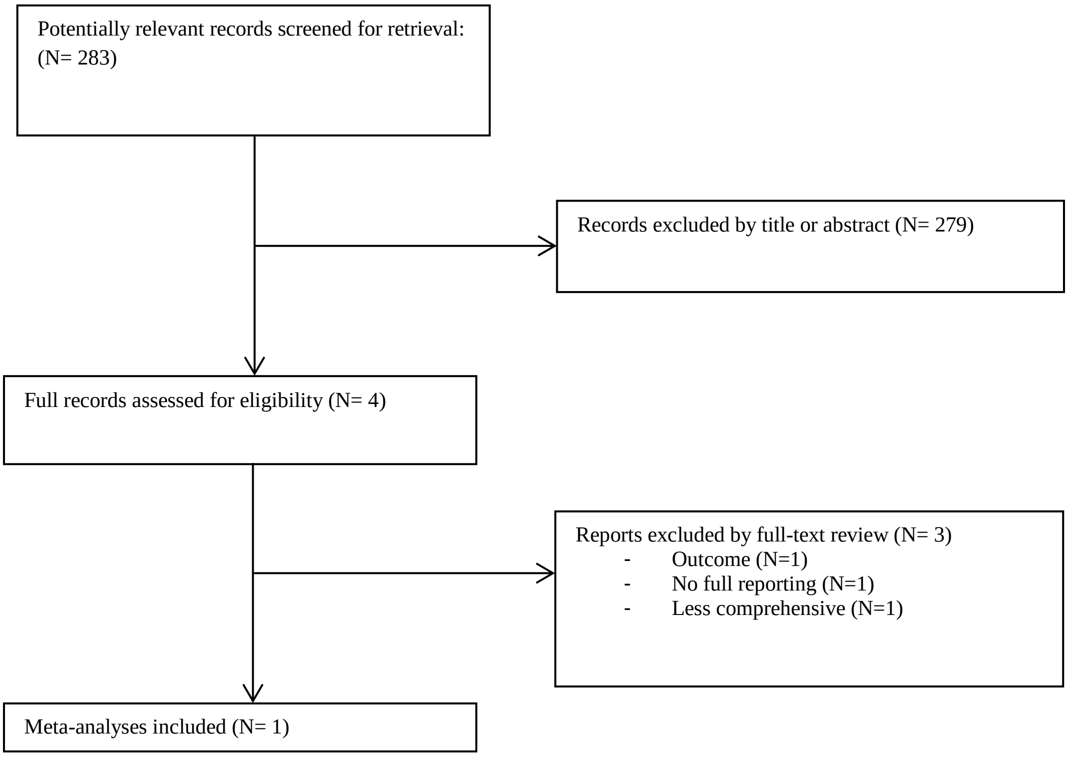


Included study:
MacLean C. Newberry S. Maglione M. et al. Systematic review: comparative effectiveness of treatments to prevent fractures in men and women with low bone density or osteoporosis. Ann Intern Med. 2008 Feb 5;148(3):197-213. Epub 2007 Dec 17.

PICO questions:

| | |
|---|---|
| People: | people with low bone density or osteoporosis |
| Intervention: | various therapies (including bisphosphonates) |
| Comparison: | placebo, with each other |
| Outcome: | rate of or risk for fractures, adverse events |

**[17] Overactive Bladder (anticholinergics)**
("urinary bladder, overactive"[MeSH Terms]) AND "meta analysis"[Publication Type]

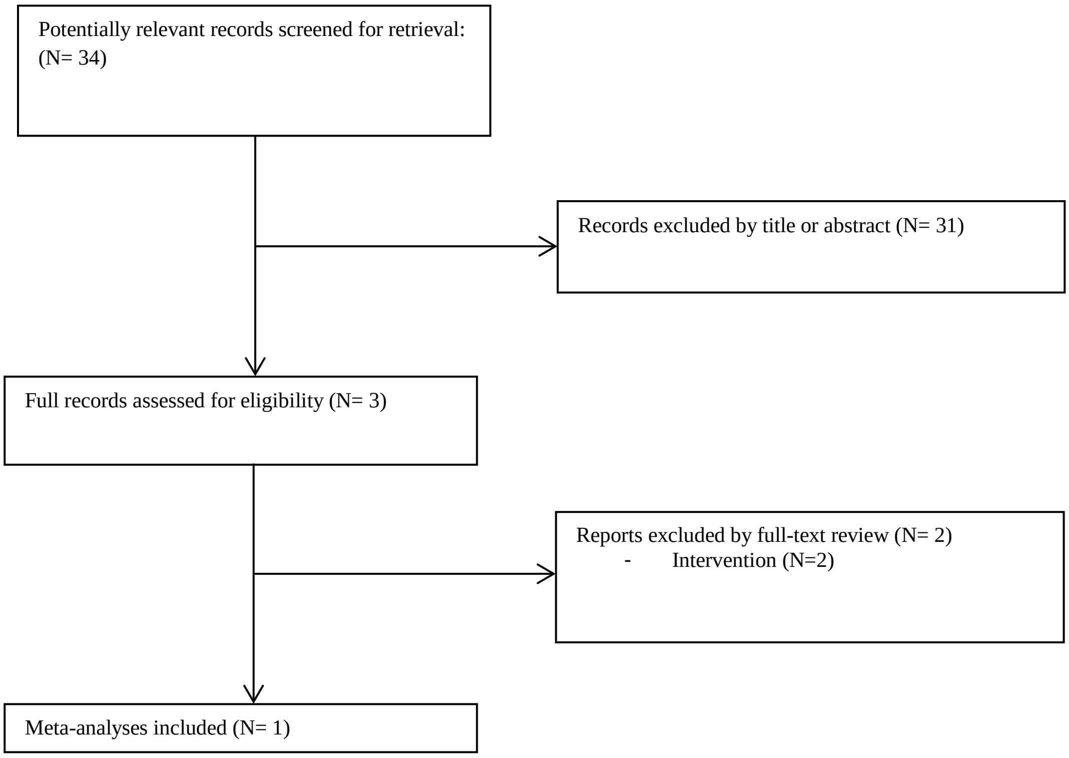


Included study:
Nabi G. Cody JD. Ellis G. Herbison P. Hay-Smith J. Anticholinergic drugs versus placebo for overactive bladder syndrome in adults. Cochrane Database Syst Rev. 2006 Oct 18;(4):CD003781.

PICO questions:

| | |
|---|---|
| People: | adults with overactive bladder syndrome |
| Intervention: | anticholinergic drug |
| Comparison: | no treatment, placebo |
| Outcome: | various patient observations, number of leakage episodes, frequency and volume, quality of life, adverse events |

**[18] Parkinson's (levodopa)**
("parkinson disease"[MeSH Terms]) AND "meta analysis"[Publication Type]

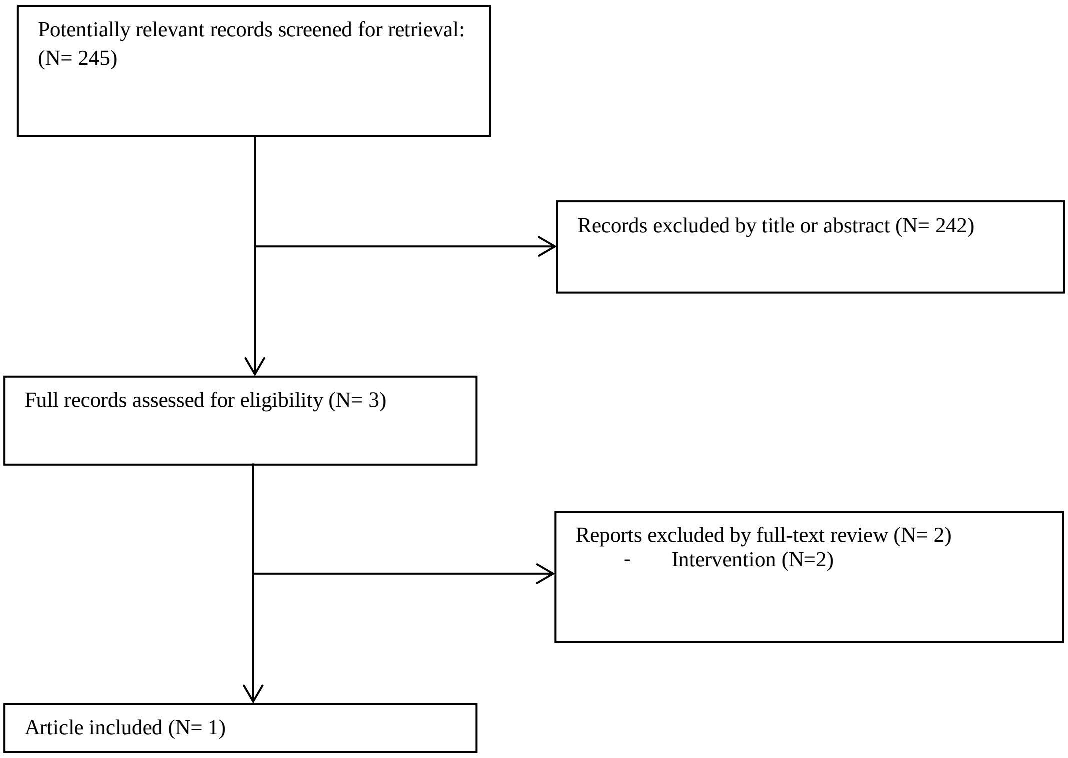


Included study:
Fahn S. Oakes D. Shoulson I. Kieburtz K. Rudolph A. Lang A. et al. Levodopa and the progression of Parkinson’s disease. N Engl J Med 2004; 351: 2498.

PICO questions:
People: patients with early Parkinson’s disease
Intervention: carbidopa–levodopa
Comparison: placebo
Outcome: reduction of symptoms

**[19] Migraine (analgesics)**
("migraine disorders"[MeSH Terms]) AND "meta analysis"[Publication Type]

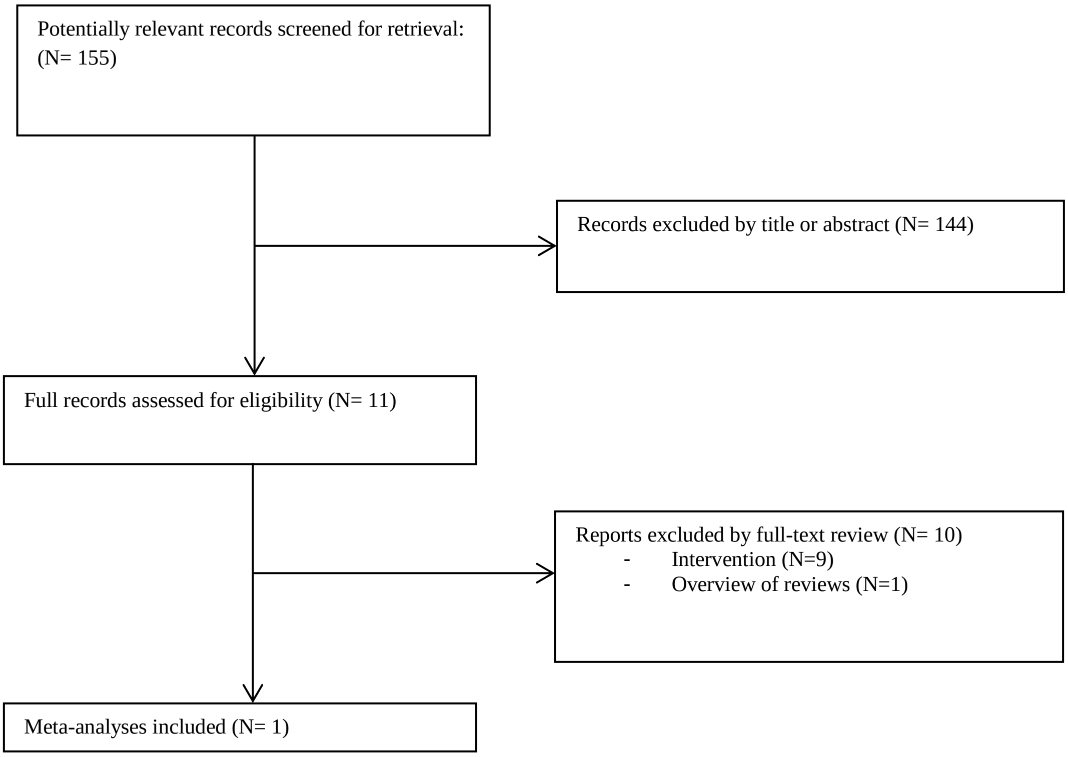


<u>Included study:</u>
Derry CJ. Derry S. Moore RA. Sumatriptan (oral route of administration) for acute migraine attacks in adults. Cochrane Database of Systematic Reviews 2012. Issue 2.

<u>PICO questions:</u>

| | |
|---|---|
| People: | adults with migraine |
| Intervention: | oral sumatriptan |
| Comparison: | placebo or other active intervention |
| Outcome: | pain-free at two hours, headache relief, sustained pain-free during the 24 hours postdose, sustained headache relief during the 24 hours postdose, pain intensity and pain relief |

**[20] Alzheimer's (cholinesterase inhibitors)**
("alzheimer disease"[MeSH Terms]) AND "meta analysis"[Publication Type]

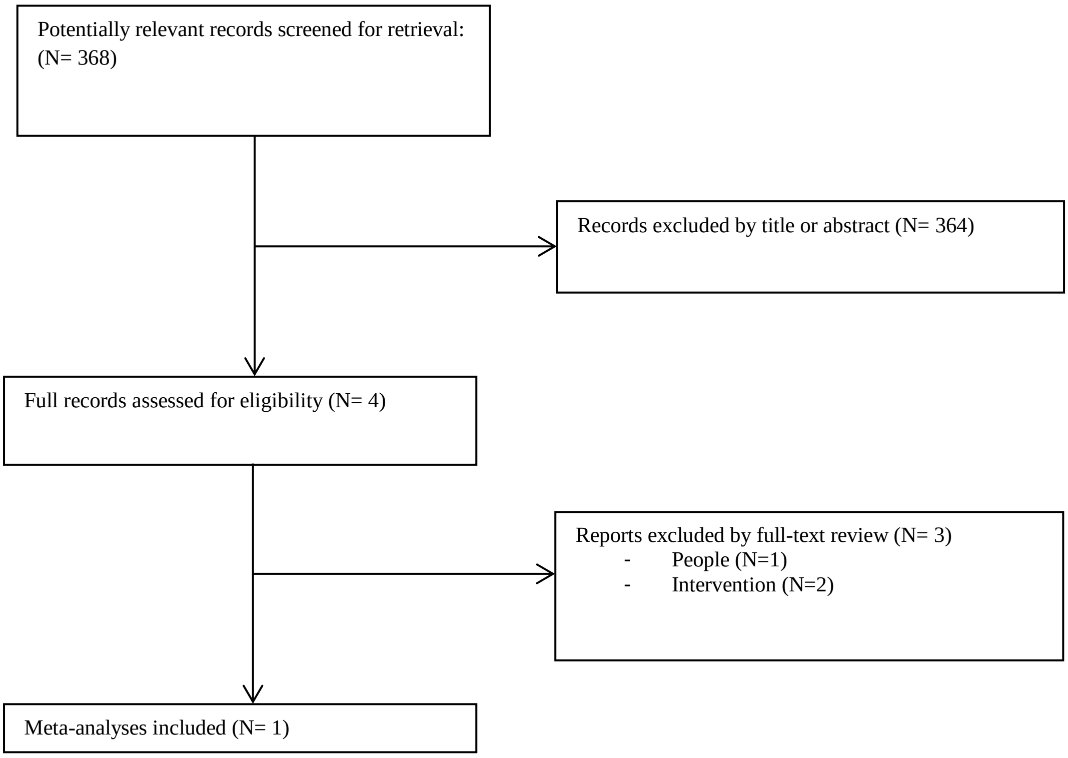


<u>Included study:</u>
Birks J. Cholinesterase inhibitors for Alzheimer's disease. Cochrane Database Syst 571 Rev. 2006(1):CD005593.

<u>PICO questions:</u>
People: people with mild, moderate or severe dementia due to Alzheimer’s disease
Intervention: cholinesterase inhibitors (donepezil, galantamine and rivastigmine)
Comparison: placebo, with each other
Outcome: reduction of symptoms
