# Supplementary material for: How effective are common medications: a perspective based on meta-analyses of major drugs
Source: BMC Med. 2015 Oct 2;13:253. doi: 10.1186/s12916-015-0494-1 (PMC4592565; doi:10.1186/s12916-015-0494-1)
Supplement: Additional file 2: — Study protocol. (PDF 161 kb) [file 12916_2015_494_MOESM2_ESM.pdf]

Additional file 2

Study protocol

PROTOCOL

How effective are common medications?
A perspective based on the 20 most frequently used drugs

Stefan Leucht, Bartosz Helfer and John M. Davis

Corresponding author:

Prof. Dr. med. Dr. h.c. Stefan Leucht
Vice chairman
Department of Psychiatry and Psychotherapy TU-München
Honorary Professor of Evidence-based Psychopharmacological Treatment
University of Aarhus

Address:
Klinikum rechts der Isar
Ismaningerstr. 22
81675 München
Germany
Tel.: +49-89-4140-4249
Fax.: +49-89-4140-4987
e-mail: Stefan.Leucht@lrz.tum.de

Background

Clinicians must often evaluate comparative risks and benefits of treatments for patients with multiple maladies. Research shows that decision making can be distorted by various cognitive biases such as a physician's tendency to remember dramatically successful cases and forget failures or misinterpret the statistical indices used in clinical trials and meta-analyses[1]. Most effort has been directed at actual statistical computation, not at training physicians to interpret results[2].

To address this issue, we prepared an evidence-based summary of the efficacy of common drugs that we believe will help physicians to more quickly and easily comprehend significant data.

Objective

To present a summary of the efficacy of the twenty most frequently used pharmacologic therapy types, as measured by the number of on-therapy patients in the US, according to the IMS Institute for Healthcare Informatics[3].

Our analysis was restricted to primary indications of these drug classes/ therapy types.

Therapy types (drug class or primary indication):
[1] Hypertension (antihypertensive drugs)
[2] Cholesterol (statins)
[3] Antidepressants (major depressive disorder)
[4] Anti-Ulcerants (proton pump inhibitors)
[5] Narcotics (analgesics for postoperative pain)
[6] Antidiabetes (metformin, GLP-1 analogues, DPP4-Inhibitors, SGLT2 inhibitors)
[7] Thyroid (thyroid preparations for hypothyreosis)
[8] Anti-Epileptics (epileptic seizures)

[9] Contraceptives (hormonal contraceptives for birth control)
[10] Respiratory (asthma, COPD)
[11] Antiplatelets/anticoagulants (acetylsalicylic acid)
[12] ADHD (Amphetamines, Methylphenidates)
[13] Insomnia (Benzodiazepines)
[14] Benign Prostate Hyperplasia (a-adrenergic blockers, 5-alpha-reductase inhibitors)
[15] Antipsychotics (schizophrenia)
[16] Osteoporosis (bisphosphonates)
[17] Overactive Bladder (anticholinergics)
[18] Parkinson's (levodopa)
[19] Migraine (analgesics)
[20] Alzheimer's (cholinesterase inhibitors)

At least 212.5 million patients are treated in 20 leading therapy areas and represent 45% of all spending and 61% of all prescriptions in 2013 in the US[3].

Method
We searched for meta-analyses on the effect of drugs monotherapy versus placebo/no treatment published in peer-reviewed journals. Studies had to include populations with a primary diagnosis of a specific disorder. There were no language or publication year restrictions. We reconstructed PICO questions[4] for each included study to ensure that they fulfil the inclusion and exclusion criteria to the fullest extent.

Inclusion Criteria:

- Systematic reviews with meta-analyses of randomized-controlled-trials
- Primary indications of drug classes/ therapy types as provided by IMS Institute for Healthcare Informatics (see ‘Objectives’)
- Monotherapy (e.g. not “antidepressant + mood stabilizer”)
- Comparators: placebo or no treatment
- Classes of drugs rather than single drugs (e.g. ACE-inhibitors, rather than only enalapril)
- We attempted to include the broadest review that was also most recent and provided the necessary data

Exclusion criteria:

- Systematic reviews without meta-analysis
- Before versus after effect sizes in contrast to between interventions effect sizes
- Special subgroups (e.g. treatment of therapy refractory schizophrenia, primary care patients, elderly)
- Meta-analyses dealing with comorbidities (e.g. diabetes and substance abuse)
- Incomplete presentation of results and missing data not calculable

Data Sources

Pubmed.

Included articles were scanned for cross-references and retrieved if suitable and not found before.

Search Strategy

Search terms combined the Medical Subject Heading Terms (MeSH) for therapy types and/or drug classes when necessary with “meta-analysis” as publication type. Some of the searches were based on our previously published analysis[5] and updated to 5 August 2014.

Outcomes

Primary indications (e.g. ACE inhibitors for hypertension and not for kidney disease) and main outcomes were used (reduction in depressive symptoms for major depressive disorder and not pain reduction).

Selection of studies and data extraction

B.H performed the update searches; B.H. and S.L. selected the reports. Data were extracted by B.H and S.L. independently verified them.

Measures of treatment effect

Whenever calculated data from intent-to-treat analysis were used.

If studies with multiple control or treating groups reported results of comparisons with placebo and control group separately, all were used to compare the effect sizes between different controls.

Dichotomous data

For binary outcomes, we extracted the percentage of patients with an outcome in each group, the risk ratio (RR), the absolute risk or response difference (ARD) and the relative risk reduction (RRR), all together with their 95% confidence intervals (CI), If no RR was reported the odds ratio was used (OR).

Continuous Data

For continuous outcomes we extracted both the mean difference (MD) and standardized mean difference (SMD) between groups after the intervention and at follow-up.

Dealing with missing data

In case no data were available we transformed the existing data to one of the standard parameters (WMD/SMD/ARD/RRR/RR), or re-calculate the meta-analyses with Comprehensive Meta-Analysis version 2[6] or Review Manager version 5.2[7].

Evaluation of results

We will tabulate the results and discuss them qualitatively.

References

1. Gigerenzer G, Muir Gray JA. Better doctors, better patients, better decisions: Envisioning health care 2020. Cambridge, MA: MIT Press; 2011.
2. Windish DM, Huot SJ, Green ML. Medicine residents' understanding of the biostatistics and results in the medical literature. JAMA. 2007 Sep 5;298(9):1010-22.
3. Medicine use and shifting costs of healthcare. A review of the use of medicines in the United States in 2013. IMS Institute for Healthcare Informatics, USA 2014, 44.
4. Huang X, Lin J, Demner-Fushman D. Evaluation of PICO as a knowledge representation for clinical questions. AMIA Annu Symp Proc. 2006:359-63
5. Leucht S, Hierl S, Kissling W, Dold M, Davis JM. Putting the efficacy of psychiatric and general medicine medication into perspective: review of meta-analyses. Br J Psychiatry. 2012 Feb;200(2):97-106.
6. Borenstein,M.,Hedges,L.,Higgins,J.,Rothstein,H.,2005. Comprehensive Meta-Analysis, 2nd ed. Biostat, Englewood, NJ.
7. Centre, T.N.C.,2012.Review Manager(RevMan), 5.2 ed. The Cochrane Collaboration, Copenhagen.
